# Phlpp1 is associated with human intervertebral disc degeneration and its deficiency promotes healing after needle puncture injury in mice

**DOI:** 10.1038/s41419-019-1985-3

**Published:** 2019-10-03

**Authors:** Changli Zhang, Madeline P. Smith, George K. Zhou, Alon Lai, Robert C. Hoy, Victoria Mroz, Olivia M. Torre, Damien M. Laudier, Elizabeth W. Bradley, Jennifer J. Westendorf, James C. Iatridis, Svenja Illien-Jünger

**Affiliations:** 1 Emory University School of Medicine, Atlanta, GA USA; 2 Icahn School of Medicine at Mount Sinai, New York, NY USA; 3 Univeristy of Minnesota, Minneapolis, MN USA; 4 Mayo Clinic, Rochester, MN USA

**Keywords:** Immunochemistry, Phosphorylases

## Abstract

Back pain is a leading cause of global disability and is strongly associated with intervertebral disc (IVD) degeneration (IDD). Hallmarks of IDD include progressive cell loss and matrix degradation. The Akt signaling pathway regulates cellularity and matrix production in IVDs and its inactivation is known to contribute to a catabolic shift and increased cell loss via apoptosis. The PH domain leucine-rich repeat protein phosphatase (Phlpp1) directly regulates Akt signaling and therefore may play a role in regulating IDD, yet this has not been investigated. The aim of this study was to investigate if Phlpp1 has a role in Akt dysregulation during IDD. In human IVDs, Phlpp1 expression was positively correlated with IDD and the apoptosis marker cleaved Caspase-3, suggesting a key role of Phlpp1 in the progression of IDD. In mice, 3 days after IVD needle puncture injury, Phlpp1 knockout (KO) promoted Akt phosphorylation and cell proliferation, with less apoptosis. At 2 and 8 months after injury, Phlpp1 deficiency also had protective effects on IVD cellularity, matrix production, and collagen structure as measured with histological and immunohistochemical analyses. Specifically, Phlpp1-deletion resulted in enhanced nucleus pulposus matrix production and more chondrocytic cells at 2 months, and increased IVD height, nucleus pulposus cellularity, and extracellular matrix deposition 8 months after injury. In conclusion, Phlpp1 has a role in limiting cell survival and matrix degradation in IDD and research targeting its suppression could identify a potential therapeutic target for IDD.

## Introduction

Back pain is a leading cause of global disability and is strongly associated with intervertebral disc (IVD) degeneration (IDD)^1^. Today’s operative and non-operative treatments for IDD have low efficacy and the current focus on painful conditions has resulted in over-reliance on opioids and is a contributor to the current opioid crisis^2^, making the development of treatments to prevent IDD a priority.

The causes for painful IDD are multifactorial and include risk factors such as heritability, lack of sport activities, complex loading, and trauma^3–6^. IDD is biochemically characterized by apoptosis, IVD structural deterioration, and extracellular matrix (ECM) degradation including glycosaminoglycan (GAG) loss and increased catabolic enzymes^6–11^. It has been demonstrated that Akt signaling regulates apoptosis and ECM degradation in IVD cells^12–14^ and inactivation of Akt likely contributes to cell loss and poor healing capacity, which are known to accelerate IDD. Regulators of Akt signaling, such as phosphatase and tensin homolog (PTEN), protein phosphatase 2 A, and calcineurin^15^, can inhibit Akt activity by controlling its upstream signaling or by dephosphorylating Akt at Thr308 and/or Ser473.

The pleckstrin homology leucine-rich repeat protein phosphatase (Phlpp1, pronounced flip) more directly targets Akt1-3 and dephosphorylate the Ser473 activation site, thereby reducing its activity^16–18^. Phlpp1 is expressed in musculoskeletal tissues^15^ where it is involved in several conditions that are implicated with IDD, such as insulin resistance, obesity, and osteoarthritis^19–22^.

In tissues with limited healing abilities, such as the heart^23^, central nervous system^24^, and cartilage^25^, reduced Phlpp1 activation can promote healing. For example, Phlpp1 depletion in brain and heart tissues enhanced Akt activation and provided neuro-^26^ and cardioprotection^27^ after injury. In murine tibia, Phlpp1 depletion limited osteoarthritis progression by increasing the cellular content of articular cartilage after destabilization of the medial meniscal tibial ligament^22^. In contrast, inhibition of Phlpp1 decreased Akt phosphorylation in adult rat hippocampus^17^ and promoted neuronal apoptosis after spinal cord injury in mice^28^. However, Phlpp1 also acts as an important tumor suppressor by terminating Akt signaling, and thus induces apoptosis and suppresses tumorigenesis in a variety of cancers^15,29,30^. Taken together, although Phlpp1 might act via similar mechanisms in different tissues, the outcomes of Phlpp1 interactions are highly context-specific and its depletion can be both physiologic and pathophysiologic.

The role of Phlpp1 in IDD has not been established. Its tight regulation of Akt signaling suggests that it plays an essential role in Akt-regulated IVD cellularity and matrix homeostasis. The aim of this study was to determine whether Phlpp1 plays a key role in IDD progression in humans and mice. We hypothesized that Phlpp1 regulates cell proliferation, apoptosis, and matrix degradation in IDD by modulation of Akt phosphorylation. Utilizing human IVD tissues and in vivo Phlpp1 KO mouse IVD injury models, we (1) investigated the correlation of Phlpp1 expression with human IDD, and (2) mechanistically evaluated the role of Phlpp1 on IVD cellularity and matrix degradation in the progression of IDD by utilizing a needle puncture mouse model.

## Results

### Phlpp1 expression positively correlated with human IDD and co-expressed with cleaved Caspase-3

**Fig. 1:**
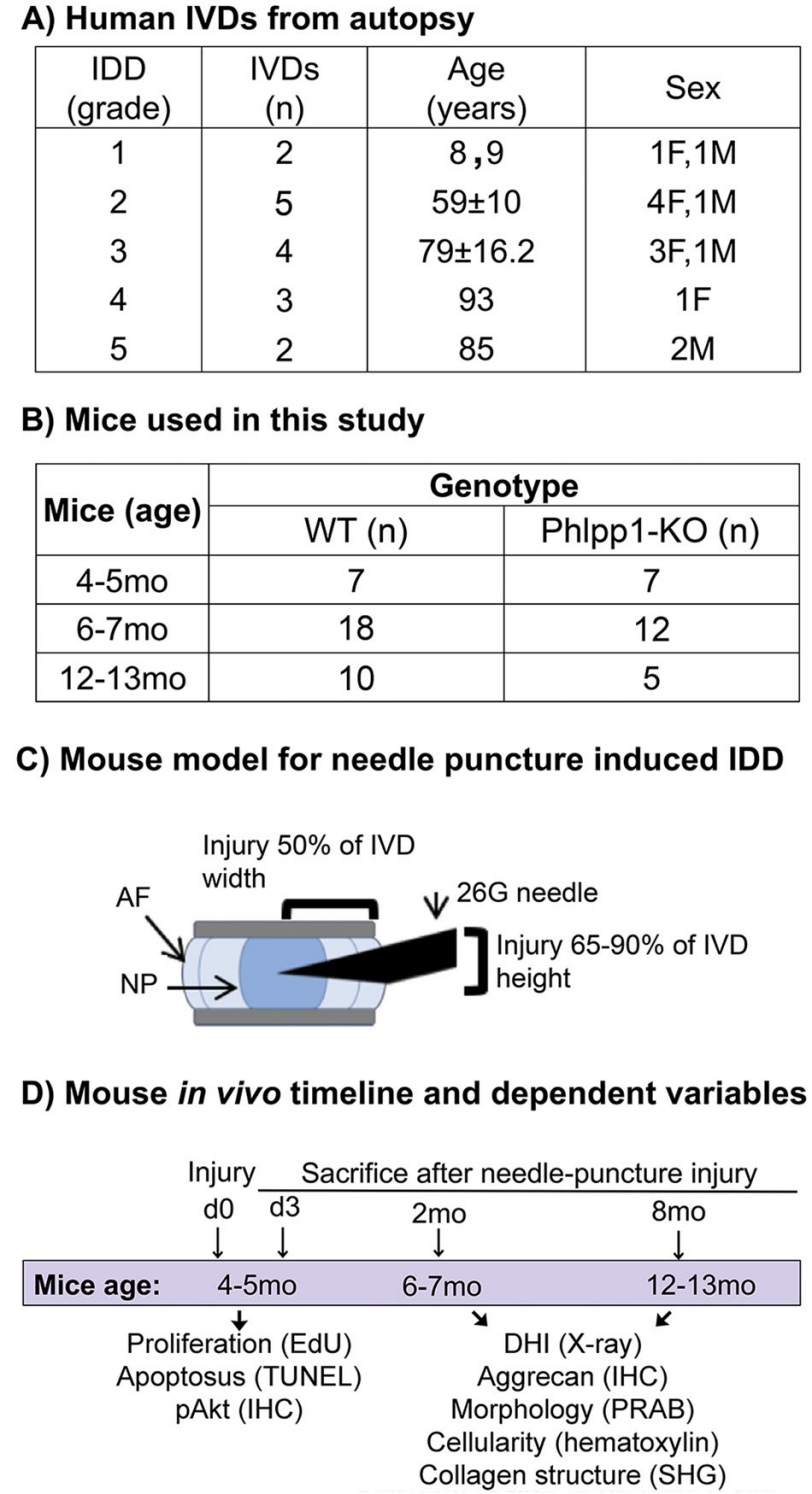
Study design. **a**) Human samples used for this study. Detailed information of human samples including degeneration grade, samples size, age, and sex. Note: three IVDs for grade 4 were collected from the same donor. **b**) The total number of mice used per timepoint and genotype. **c**) Needle puncture induced IDD mouse model. Note: due to limited availability of suitable sections showing needle track, not all experiments could be performed on each mouse. The exact number of mice per experiment is stated in the corresponding figure legend. **d**) Timepoints and dependent variables in mouse model study

**Fig. 2:**
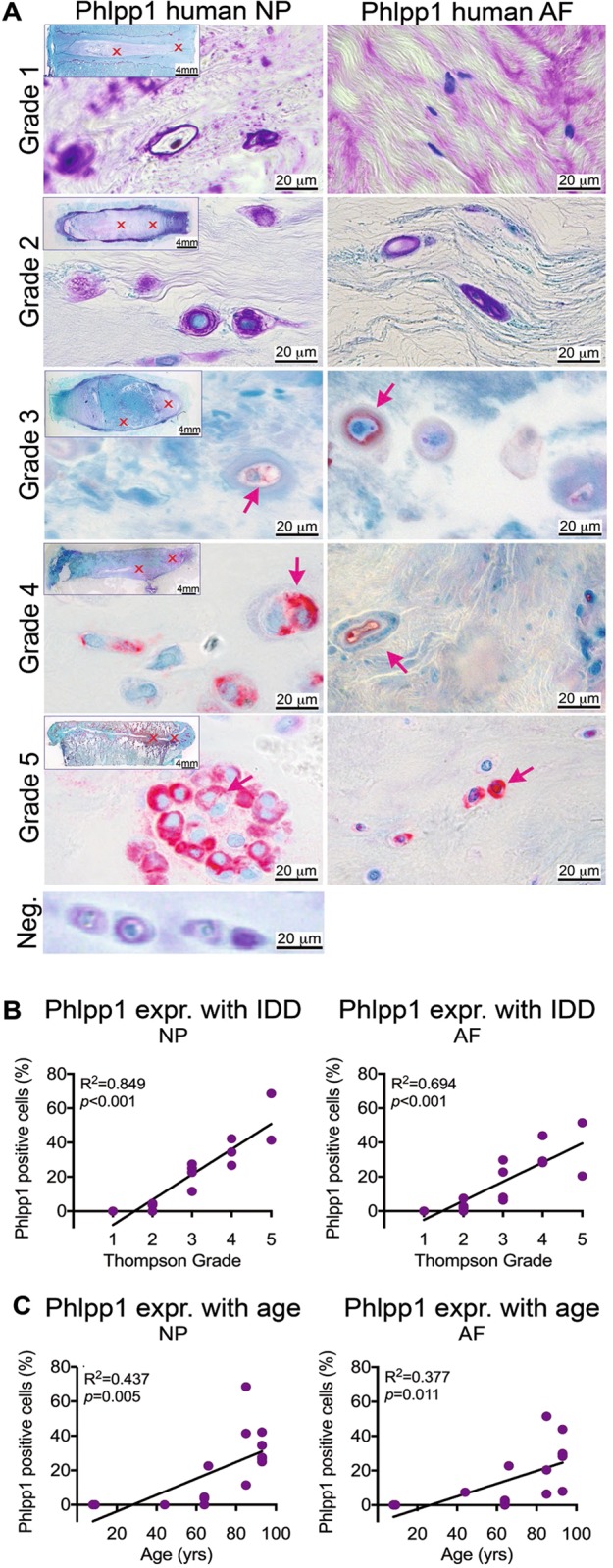
Phlpp1 is positively correlated with human intervertebral disc degeneration. **a**) Representative images of immunohistochemistry staining for Phlpp1 (red) in human IVDs with degeneration grade 1–5. Left: Phlpp1 stain in the NP, right: Phlpp1 stain in the AF. Arrows indicate positive cells. Neg.: negative control of Phlpp1 stain with primary antibody being replaced by negative control IgG. **b**+**c**) Quantification of staining for Phlpp1 and its correlation with **b**) degeneration grade and **c**) age. Expression of Phlpp1 was strongly correlated with the degeneration grade and to a lesser extent with age both in the NP and AF (*n* = 16)

To investigate the association of Phlpp1 expression with human IVD aging and degeneration, we collected human IVDs from autopsy of varying age and degeneration grades (Fig. 1a). In degenerated IVDs, Phlpp1-positive cells were frequently observed in areas close to fissures within the NP and AF (Fig. 2a, red stain). Little positive staining was observed in grade 1 and 2 IVDs, with greater staining in moderate (grade 3) IDD and strong staining in severe (grades 4 and 5) IDD (Fig. 2b), indicating a strong and positive correlation of Phlpp1 expression with IDD within the NP (*R*^2^ = 0.849; *p* < 0.001) and AF (*R*^2^ = 0.694; *p* < 0.001). In contrast, Phlpp1 expression was weakly correlated with aging in both NP (*R*^2^ = 0.437; *p* = 0.005) and AF (*R*^2^ = 0.377; *p* = 0.011) tissues (Fig. 2c).

**Fig. 3:**
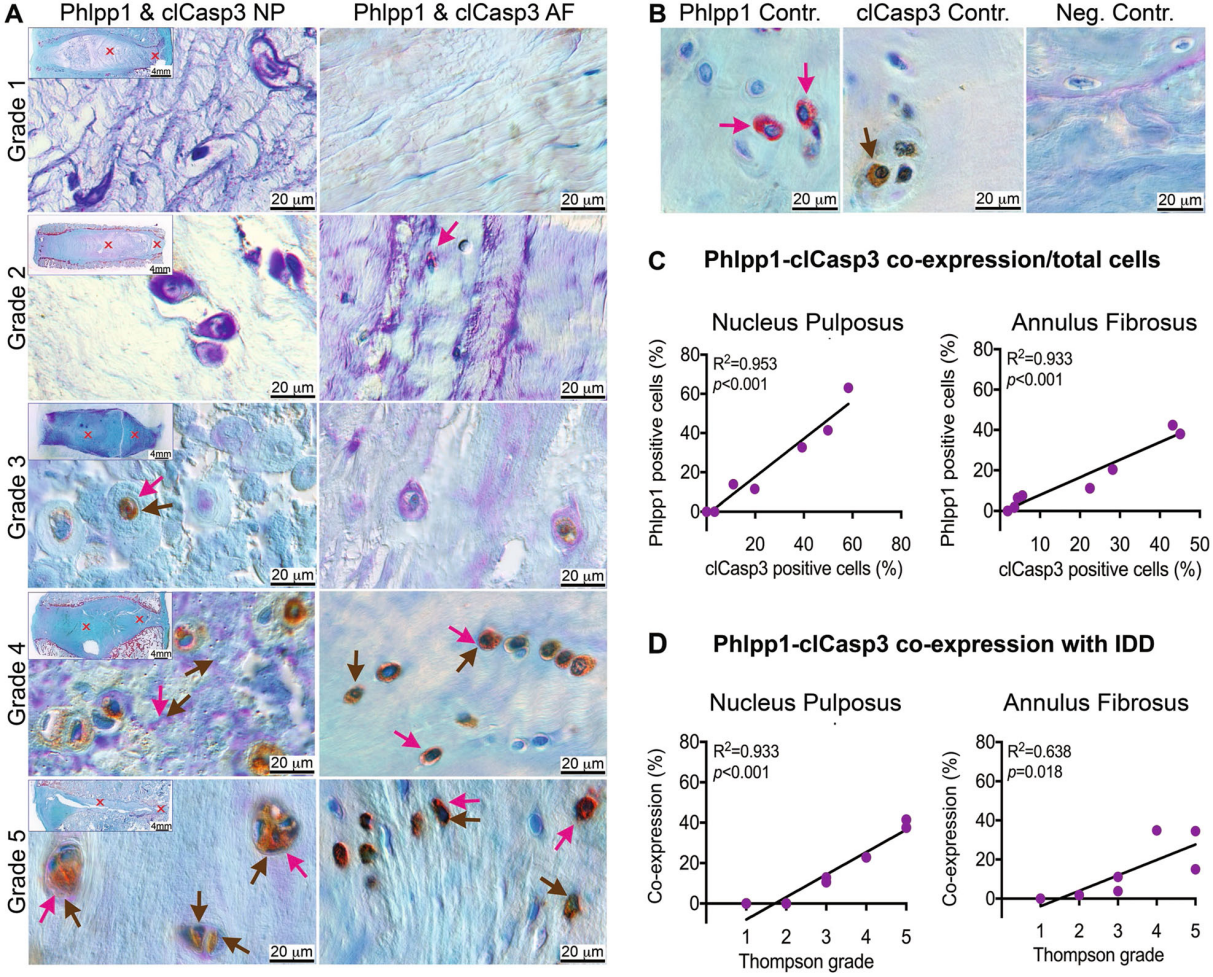
Phlpp1 and clCasp3 are often co-expressed and increased with IVD degeneration. **a**) Representative DIC images for the co-expression of Phlpp1 (red) with clCasp (brown). Red and brown arrows indicate positive cells for Phlpp1 and clCasp3, respectively. DIC: differential interference contrast. **b** Single positive stain for Phlpp1 (red) and clCasp3 (brown). Neg.: negative control of Phlpp1 stain with primary antibody being replaced by negative control IgG. **c**+**d** Quantification of Phlpp1-clCasp co-expression and its correlation with degeneration grade (*n* = 8). Phlpp1 was strongly correlated with clCasp3 expression both in the NP and AF. Their co-expression was positively correlated with the degeneration grade in the NP, and to a lesser extent in the AF

Human IVD aging and degeneration are often associated with increased cell apoptosis, as demonstrated by increased TUNEL-positive cells and apoptosis executioner cleaved Caspases-3 (clCasp3)^11,31,32^. To determine whether Phlpp1 was associated with apoptosis, we assessed the co- expression of Phlpp1 and clCasp3 (Fig. 3a–d). A strong correlation between Phlpp1- and clCasp3-positive cells were detected in both NP (*R*^2^ = 0.953; *p* < 0.001) and AF (*R*^2^ = 0.933; *p* < 0.001) regions (Fig. 3e). The proportion of cells co-expressing Phlpp1 and clCasp3 was increased with varying degeneration grades in both NP (*R*^2^ = 0.933; *p* < 0.001) and AF (*R*^2^ = 0.638; *p* = 0.018) tissues (Fig. 3d), suggesting Phlpp1 as an important contributor to the progression of human IDD via apoptosis.

### Phlpp1 depletion promoted Akt phosphorylation 3 days after needle puncture injury

**Fig. 4:**
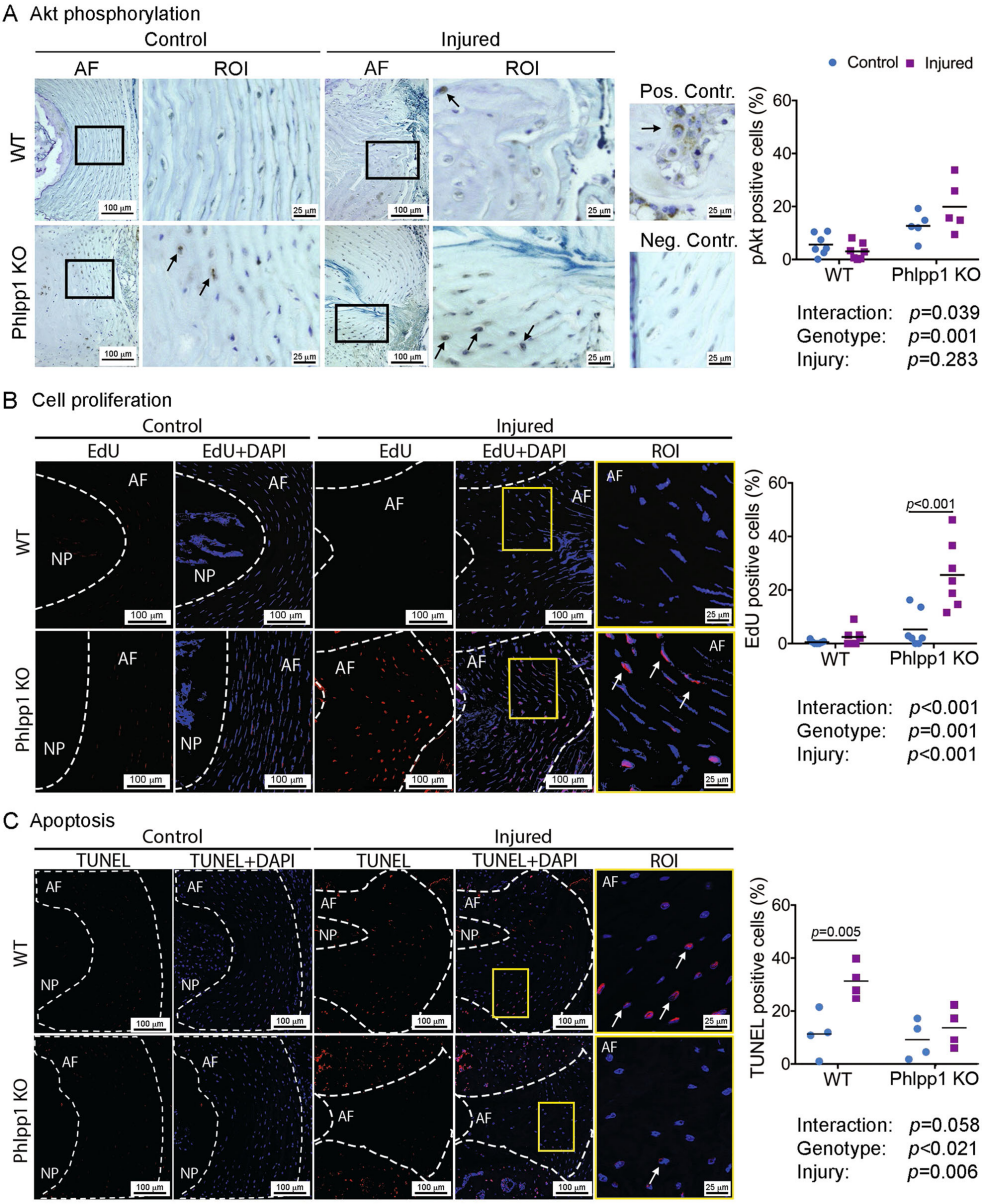
Phlpp1-deletion (A) promoted Akt phosphorylation, (B) increased cell proliferation, and (C) decreased apoptosis 3 days after needle puncture injury. **a**) Immunohistochemistry staining for pAkt demonstrated elevated Akt phosphorylation in the AF of Phlpp1 KO (*n* = 5) compared to WT mice (*n* = 7) at baseline. Injury further caused an increase of pAkt in Phlpp1 KO mice, but not in WT mice 3 days after needle puncture. ROI (region of interest) is the magnification of the region in the black boxes. Arrows indicate positive cells. Positive and negative controls were used for antibody validation. **b**) EdU and **c**) TUNEL assay of IVD sections 3 days after needle puncture. EdU-positive cells were increased in injured AF of Phlpp1 KO mice, compared to the other three groups (*n* = 7 per group). TUNEL-positive cells were increased significantly in AF tissues of WT mice, but not in Phlpp1 KO mice 3 days after injury (*n* = 4 per group). White dotted lines denote the border between the NP and AF compartments. Yellow boxes mark the magnified ROI. Arrows denote positive cells. Nuclei were stained with DAPI. Two-way ANOVAs were used to assess the effect of injury and genotype

To investigate the role of Phlpp1 on Akt signaling during IVD injury, we performed needle punctures in tail IVDs of 4–5 month-old C57BL/6 J wild type (WT) and Phlpp1 KO mice and assessed Akt phosphorylation 3 days after injury (Fig. 1b–d). The severe needle puncture injury often resulted in complete loss of the NP and therefore acute cellular responses were examined in AF tissues only. Immunohistochemical analysis demonstrated an increased baseline phosphorylation of Akt in Phlpp1 KO compared with WT IVDs, indicating a role of Phlpp1 in regulating basal Akt activity (Fig. 4a). Needle puncture injury caused a pronounced increase in pAkt-positive cells within Phlpp1 KO mice, but not in WT mice, suggesting that Phlpp1 deficiency accentuated Akt activation in response to acute injury.

### Phlpp1 depletion induced cell proliferation 3 days after needle puncture injury

Three days after needle puncture, AF cell proliferation was significantly increased in injured Phlpp1 KO IVDs compared to all other groups while only minor cell proliferation was observed in control Phlpp1 KO and WT mice, or in injured WT mice (Fig. 4b). Phlpp1-deletion decreased apoptosis 3 days after needle puncture injury.

As expected, needle puncture injury significantly increased AF cell apoptosis in injured WT IVDs (Fig. 4e). In contrast, injury did not significantly increase apoptosis in IVDs from Phlpp1 KO mice, suggesting that Phlpp1 deficiency mitigated the extent of apoptosis 3 days after needle puncture injury.

### Phlpp1 deletion prevented the continuous loss of IVD height 8 months after needle puncture injury

**Fig. 5:**
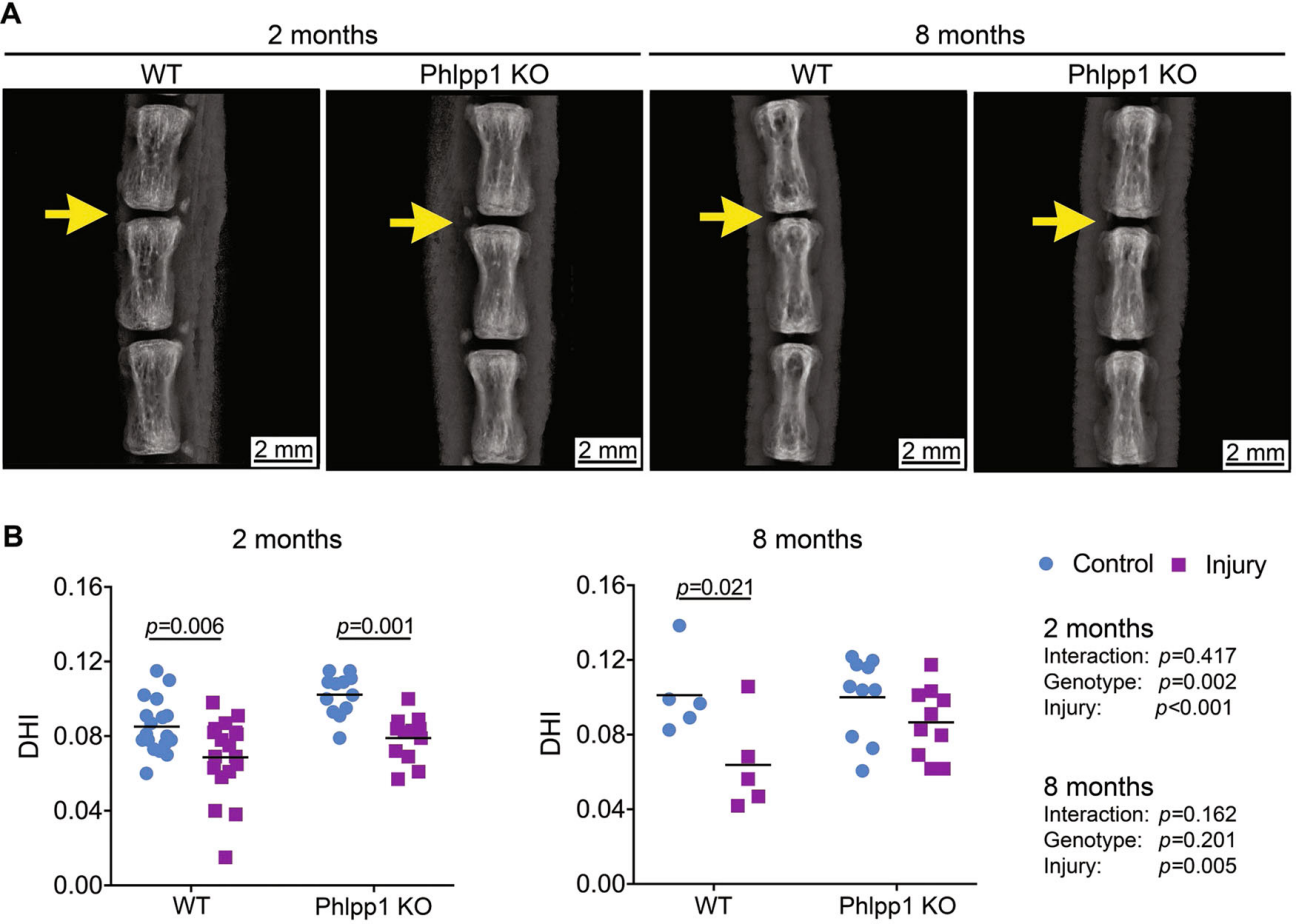
Phlpp1-deleption prevented the continuous loss of IVD height 8 months after injury. **a**) representative X-ray images of tail spines 2 and 8 months after injury. Yellow arrows indicateinjured IVD levels. **b**) DHI quantification in IVD sections. DHI was decreased both in WT (*n* = 18) and Phlpp1 KO (*n* = 12) mice 2 months after injury. By 8 months, the DHI was maintained in Phlpp1 KO mice (*n* = 10) after injury, but not in WT mice (*n* = 5). Two-way ANOVAs were used to assess the effect of injury and genotype

To assess the long-term effects of Phlpp1 in needle puncture induced injury, we examined the changes in disc height, a sensitive marker of IVD injury, 2 and 8 months after injury (Fig. 5). We calculated the disc height index (DHI) by normalizing the IVD height to vertebrae height to account for natural variation of body size between mice. Quantification of X-ray images demonstrated significantly decreased DHI 2 months after needle puncture injury, which was independent of genotype. However, at 8 months, there was no significant difference in DHI between injured and control Phlpp1 KO IVDs, whereas DHI remained significantly decreased in injured compared with control WT IVDs. These results suggested that Phlpp1-deletion prevented the progression of continuous IVD height loss after needle puncture injury.

### Phlpp1-deletion improved NP cellularity after needle puncture injury

**Fig. 6:**
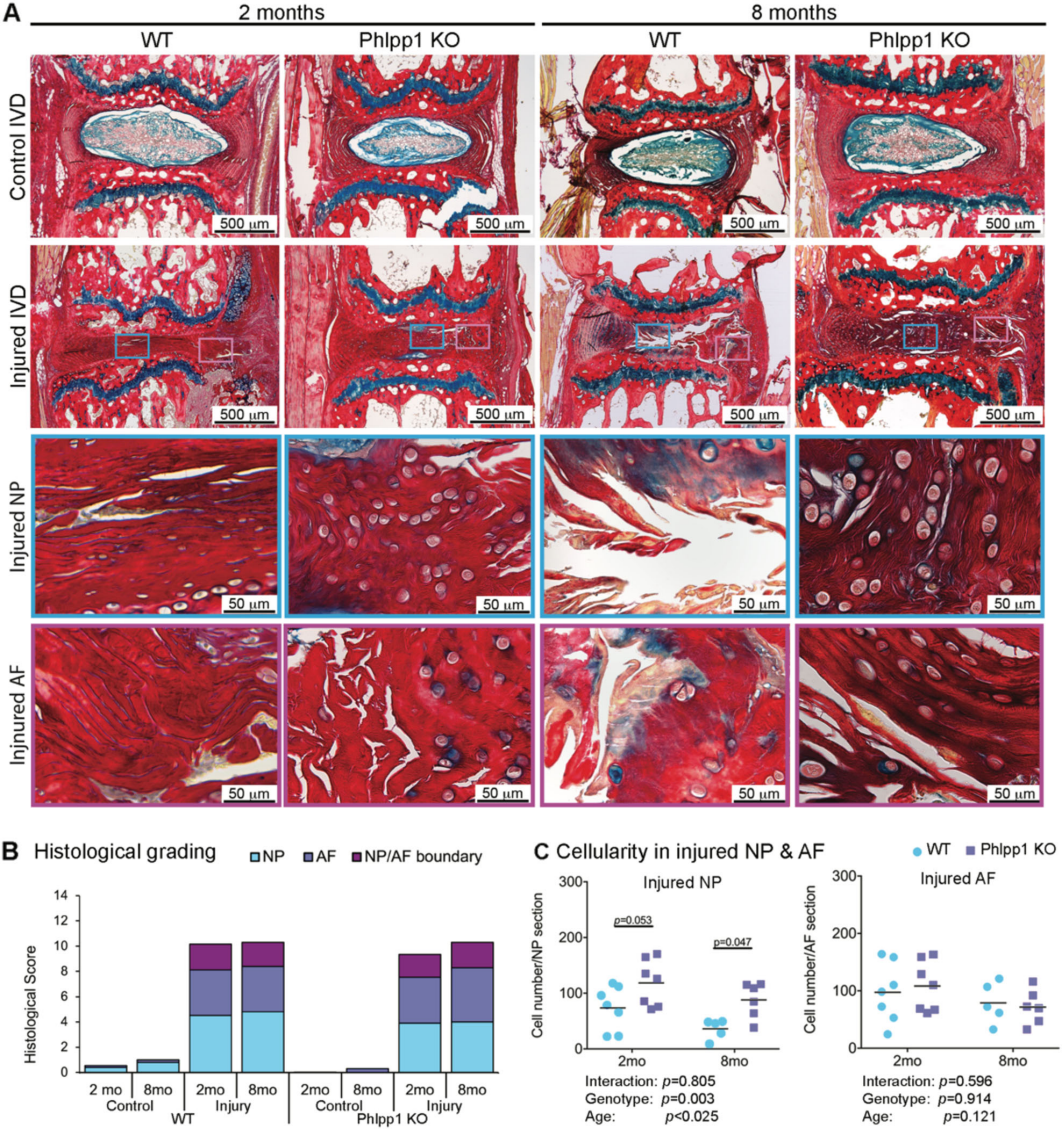
Phlpp1-deletion improved NP cellularity after needle puncture injury. **a**) Representative images of Picrosirius Red- Alcian Blue staining of IVD sections 2 and 8 months after injury. Green boxes indicate ROI of the NP and pink boxes indicate the AF. **b**) Distribution of IVD score of the NP, AF, and NP/AF boundary. IVD score was significantly increased both in WT and Phlpp1 KO at 2 (*n* = 7 for control WT, *n* = 6 for injured WT mice; *n* = 10 for control Phlpp1 KO and *n* = 8 for injured Phlpp1 KO mice) and 8 months (*n* = 5 per group) after injury, but no differences were observed between injured WT and Phlpp1 KO IVDs at both timepoints. **c**) Cellularity in NP and AF tissues after injury. The injured NP of Phlpp1 KO mice tended to be more cellular than the cellularity in WT mice both 2 (*n* = 7 per group) and 8 (*n* = 5 for WT; *n* = 6 for Phlpp1 KO) months after injury, and the cellularity of injured AF was similar between WT and Phlpp1 KO mice. Two-way ANOVAs were used to assess the effect of injury and genotype

Picrosirius Red-Alcian Blue staining was performed to examine structural alterations in IVDs 2 and 8 months after needle puncture (Fig. 6). Both Control WT and Phlpp1 KO IVDs contained a GAG-rich NP and the AF consisted of concentric lamellae of aligned collagen fibers (Fig. 6a).

In injured IVDs, despite the severe loss of NP tissue 3 days after needle puncture, the NP region was filled with a collagen-rich ECM often containing chondrocyte-like cells at 2 months, suggesting an attempted healing response in both WT and Phlpp1 KO mice. However, the intense Picrosirius Red staining together with diminished Alcian Blue intensity suggested a loss of the GAG-rich matrix within the NP. Structurally, in both injured WT and Phlpp1 KO IVDs, the AF lamellae were bulged inwards and disorganized with a blurred AF-NP boundary (Fig. 6a).

Eight months after needle puncture, structural defects accumulated further in injured WT IVDs, as demonstrated by pronounced cell loss in the NP and large fissures with fibrotic scar formation in both NP and AF tissues. In Phlpp1 KO mice, the collagenous matrix in the NP was maintained and the AF structure was not restored (Fig. 6a).

To quantitatively assess the histological changes, we used a newly established scoring system by Tam et al.^33^. The total histological score was significantly increased both in injured WT and Phlpp1 KO mice, and was not restored over time (Fig. 6b). No differences were observed between injured WT and Phlpp1 KO IVDs, neither in the total score nor within each scoring category. However, NP cellularity was increased in injured Phlpp1 KO compared to WT IVDs 2 and 8 months after needle puncture, while no differences were observed in AF cellularity (Fig. 6e). Together, these findings suggested that, while there were no differences in histological degeneration score between the two genotypes, Phlpp1 deficiency improved NP cellularity which facilitated the improvement in IVD height and prevented the further deterioration of IVD structure.

### Phlpp1-deletion improved collagen structure in the NP following needle puncture injury

**Fig. 7:**
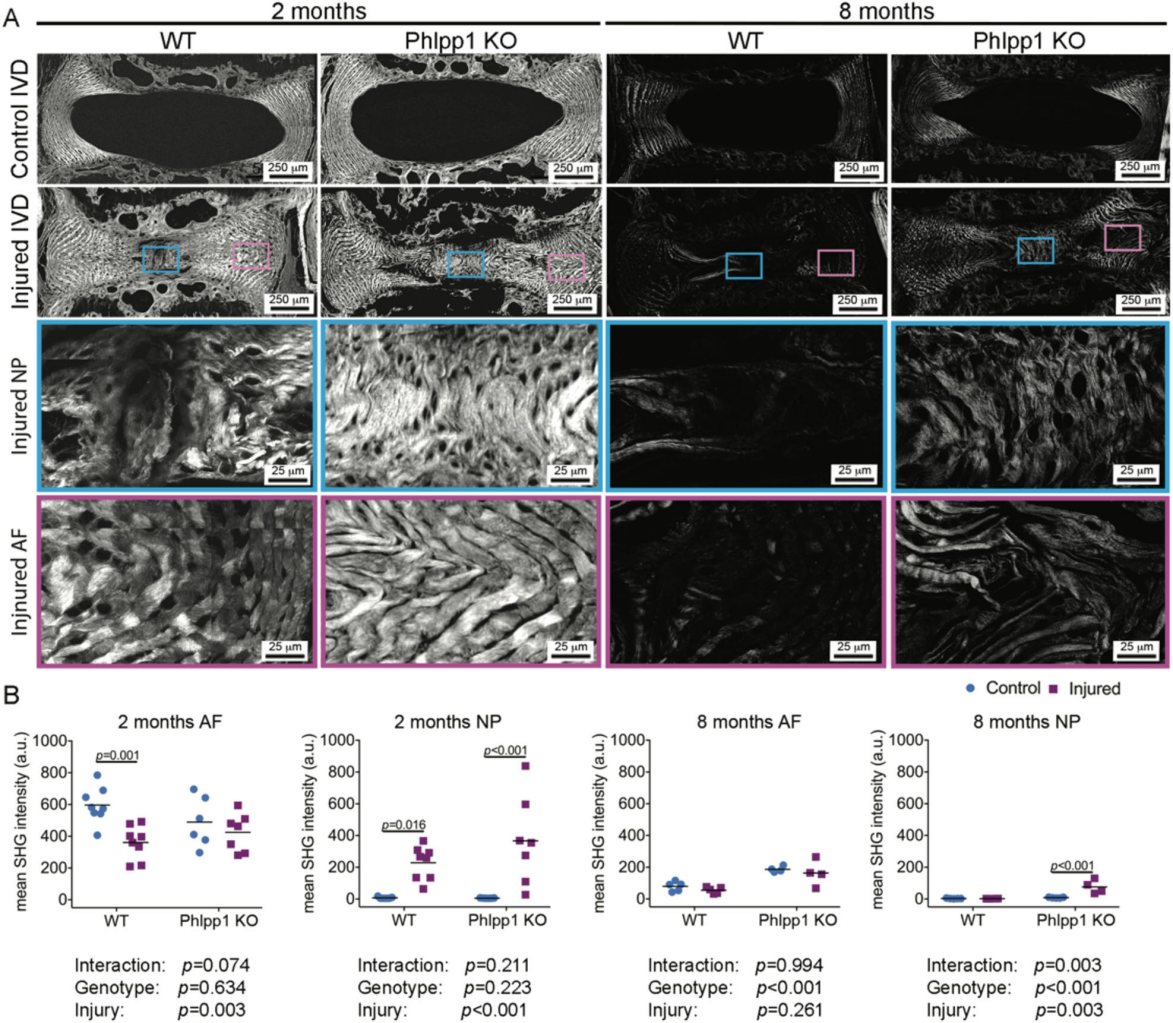
Phlpp1-deletion improved collagen structure in the NP following needle puncture injury. **a**) Representative images of SHG microscopy. Boxes indicate the ROI in injured levels of the NP (blue boxes) and the AF (pink boxes). **b** Quantification of mean SHG intensity (*n* = 7 per group at 2 months; *n* = 5 for WT and *n* = 4 for Phlpp1 KO at 8 months). Mean SHG intensity was reduced in WT 2 months after needle puncture, but not in Phlpp1 KO mice, whereas in the NP, it was increased in both WT and Phlpp1 KO mice. By 8 months, the general mean SHG intensity was decreased in all groups relative to the 2 months timepoint. The AF in Phlpp1 KO mice showed higher SHG intensity than the AF in WT mice and retained the tendency after injury. In the NP, SHG intensity was only increased in injured Phlpp1 KO mice, compared with all other three groups. Two-way ANOVAs were used to assess the effect of injury and genotype

Collagen microstructure in IVDs was assessed using second harmonic generation (SHG) imaging. In control IVDs of both WT and Phlpp1 KO mice, strong SHG signals were observed within AF tissues, whereas NP tissues had no or little SHG signal (Fig. 7a). Two months after injury, the SHG intensity was significantly reduced in injured AF tissues of WT mice, whereas no obvious changes were observed between control and injured AF tissues of Phlpp1 KO mice (Fig. 7b). In NP tissues of injured IVDs of both WT and Phlpp1 KO mice, SHG intensity significantly increased 2 months after injury. Our SHG findings of collagenous matrix infiltration into the NP region after needle puncture injury were consistent to our observations in Picrosirius Red-Alcian blue staining.

Eight months after needle puncture injury, SHG intensities in AF tissues of WT and Phlpp1 KO mice were generally reduced when compared with the 2 month timepoint. Although control and injured AF tissues exhibited similar level of the SHG intensity in both genotypes, the SHG intensity in AF tissues was higher in Phlpp1 KO than WT mice at baseline. In the NP, enhanced SHG intensity was detected in Phlpp1 KO mice but not in WT mice. These findings demonstrated that Phlpp1 deficiency protected against the progression of degenerative changes in the IVDs after injury.

### Phlpp1 deletion promoted aggrecan deposition 8 months after needle puncture injury

**Fig. 8:**
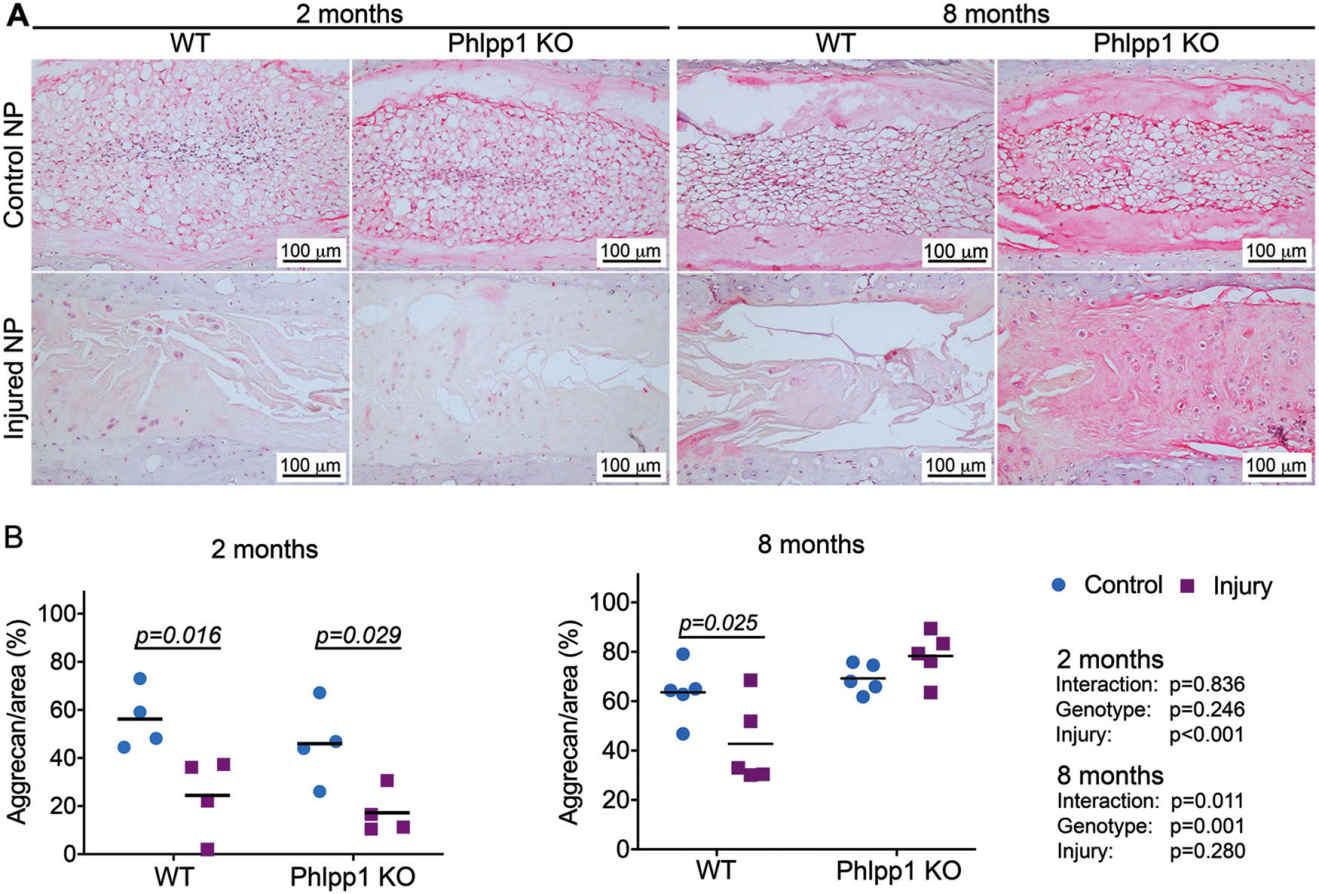
Phlpp1-deleption promoted aggrecan deposition 8 months after injury. **a**) Representative images of immunohistochemistry staining for aggrecan 2 and 8 months after needle puncture. **b**) Quantification of aggrecan-positive area. Aggrecan-positive area decreased significantly both in WT and Phlpp1 KO mice 2 months (*n* = 4 per group) after injury. By 8 months (*n* = 5 per group), the NP showed similar percentage of positive area between control and injured Phlpp1 KO mice, but it was still decreased significantly in WT mice after injury. Two-way ANOVAs were used to assess the effect of injury and genotype

To better understand the NP matrix composition of injured IVDs 2 and 8 months after injury, aggrecan content was assessed immunohistochemically. In control NP tissues of WT and Phlpp1 KO IVDs, aggrecan was deposited uniformly throughout the NP matrix except for the notochordal band. Needle puncture injury resulted in significantly decreased aggrecan content in NP tissues of both genotypes at 2 months (Fig. 8a). However, 8 months after needle puncture injury, the NP of injured Phlpp1 KO IVDs appeared homogeneously distributed and contained chondrocyte-like cells, indicating the formation of fibrocartilaginous structure. Aggrecan quantification demonstrated that the NP tissues of injured Phlpp1 KO mice exhibited more aggrecan-positive area compared with NP tissues of injured WT mice (Fig. 8b). The results suggested that Phlpp1 KO facilitated aggrecan production 8 months after injury.

## Discussion

This study revealed an association between Phlpp1 expression and human IVD degeneration, and demonstrated that Phlpp1 depletion could prevent the progression of IDD after needle puncture injury in mice. In human IVDs, our data showed a strong and positive correlation of Phlpp1 with IDD and apoptosis, a known contributor of cell loss during IDD^32,34,35^. In an acute response to injury, Phlpp1 depletion elevated Akt phosphorylation and cell proliferation while suppressing apoptosis. Over time, Phlpp1 KO promoted IVD healing from the puncture injury as indicated by improved DHI, increased NP cellularity, and matrix production 8 months after injury. Together, our data suggested that Phlpp1 is involved with the progression of IDD, and that it plays an important role in regulating cellularity and matrix accumulation in the IVD during healing of injuries via the Akt pathway.

Phlpp1 selectively inhibits cell survival throughout the suppression Akt signaling by dephosphorylating Akt2 and Akt3 at Ser473^16^. Aberrant expression of Phlpp1 has been reported in several diseases that depend on intact Akt signaling^36^, such as osteoarthritis^22^, diabetes^37^, or myocardial ischemia^38^. Dysregulated Akt signaling affects multiple downstream effectors involved in cell survival and proliferation^39^. Our data demonstrated that Phlpp1 depletion led to enhanced basal Akt phosphorylation in mice IVDs, which was further elevated in response to needle puncture injury where it increased cell proliferation and suppressed apoptosis. In vitro, enhanced Akt activity can attenuate NP cell apoptosis following mechanical overloading^14^, pro- inflammatory cytokine exposure^40^, or hyperosmotic stress^41^. In adipose and skeletal muscles and the heart of obese patients, impaired Akt activity induced by high levels of Phlpp1 limited glycogen synthesis^20^ and facilitated cardiomyocyte death^27^. Phlpp1 depletion in brain and heart tissues increased basal levels of Akt activation and cell survival facilitating neuroprotection and cardioprotection after injury, which were attenuated by Akt inhibition^37–39^. This broad literature highlighting increased cell survival with Phlpp1 depletion supports our data and together suggests a role of Phlpp1 in regulating IVD cell survival potentially via Akt signaling during IDD.

Consistent with previous studies, severe needle puncture injuries caused immediate NP herniation^42–44^ and resulted in the formation of large fissures and fibrotic tissue accumulation within the NP and AF regions 2 month after injury^42^. Although the acute response 3 days after injury did not resemble natural IDD in humans, the injury induced degenerative changes with further diminished tissue integrity, severe matrix breakdown, and cleft formation 8 months after injury, which simulated the natural progression of IDD.

Annular needle puncture injuries can cause an imbalance between catabolic and anabolic activity in IVDs by upregulating MMP3, and downregulating BMP-2 and -7^45^. In line with our findings, in a post-traumatic osteoarthritis mouse model, Phlpp1 depletion protected against the progression of osteoarthritis by increasing cellular content and attenuating cartilage degradation^22^. In human IDD, unregulated proliferative cells express a high level of catabolic enzymes MMPs and ADAMTs, leading to accumulation of degradative matrix, which attenuates protein renewal and resistance to compression^46,47^. Cleaved aggrecan fragments in degenerated IVDs promote further degeneration by lowering the osmotic pressure, ultimately leading to a loss of IVD height^48^. In our model, Phlpp1 KO prevented the progression of DHI loss and IVD matrix breakdown, likely by accumulation of aggrecan 8 months after injury compared with WT mice. However, the SHG intensity within the AF was generally decreased in 12 months old WT and Phlpp1 KO mice. A recent study by Xiao et al.^49^ demonstrated a strong correlation between AF collagen damage and age by using a collagen-hybridizing peptide (which integrates into damaged collagen) and observed a significant increase in damaged collagen between 6–8 months and 10–12 months old mice. Given that the damage to the secondary and tertiary protein structure of collagen is also detectable by decreased SHG intensity^50^, their findings supported the lower SHG intensity detected in the AF tissues of 12 months old mice relative to 6 months group. In addition, SHG imaging demonstrated increased collagenous matrix in the NP of injured Phlpp1 KO mice, indicating that Phlpp1 depletion mitigated the catabolic shift.

For this study we used a global Phlpp1 KO mouse model. We cannot exclude that the systemic depletion of Phlpp1 affected the IVD healing response in Phlpp1 KO mice by altering surrounding tissues such as tendons, ligaments, or muscles. However, we did not observe any morphological differences when comparing mature WT and Phlpp1 KO vertebral structures, suggesting that global Phlpp1-deletion had no or only minimal effects on spinal structures. The use of a global knockout allowed us to assess the effect of Phlpp1 deficiency on the entire IVD, which is composed of three distinct tissues with NP cells originating from the notochord, whereas AF and endplate cells originate from the sclerotome^51^. Using a conditional knockout model would allow targeting only a single marker, and to our knowledge no specific markers exist that are uniquely expressed in both NP, AF, and endplate cells, without being present in surrounding tissues. One limitation of using mouse IVDs to study IVD degeneration is that mice maintain a subpopulation of notochordal cells throughout life while human IVDs lose their notochordal cells during adolescence. Yet, the needle puncture resulted in complete herniation of the notochordal cell rich NP 3 days after injury. Although we are not able to study the direct effect of Phlpp1 depletion on NP cells, the positive correlation of Phlpp1 with IVD degeneration and apoptosis strongly suggests a role of Phlpp1 in human NP and AF during IDD, thus future research is warranted to verify the protective role of Phlpp1 deficiency in human NP cell culture studies.

Besides Akt, Phlpp1 also directly controls other cell survival signaling pathways, including PKC and p70 S6 kinase^15,52,53^ and the pro-apoptotic kinase Mst1^54,55^, which likely explains the protective effect of Phlpp1 depletion on cell proliferation but less pronounced effects on Akt phosphorylation. Phlpp1 deficiency also activates the negative feedback regulation of the Akt pathway, where PTEN, a lipid phosphatase, participates in the feedback loop by dephosphorylating PI3K to balance the level of phosphorylated Akt^29,49^. PTEN is an another phosphatase that has several off-target effects. One of its important functions is participating in the repair and response of DNA damage^56^. PTEN-deficient neurons exhibit seizures, autism-related disorders, and are highly prone to cancer, which do not emerge in Phlpp1-deficient mice^29^.

In summary, Phlpp1 was associated with increased human IVD degeneration and increased cleavage of caspase-3. To mechanistically identify the role of Phlpp1, a global Phlpp1 KO mouse model was used. Depletion of Phlpp1 activated Akt signaling, enhanced cell proliferation, and suppressed apoptosis, which likely promoted increased cellularity and cartilaginous matrix deposition in the NP region, preventing further progression of IDD. Small molecule Phlpp inhibitors have been synthesized and confirmed to improve cell survival and promote matrix production^57,58^. As Phlpp1 expression was increased with degeneration grades in human IVDs, local administration of Phlpp inhibitors via suitable biomaterials in herniated or mildly and moderately degenerated IVDs would alleviate the continued deterioration of IVD matrix and enhance cell survival via AKT signaling. Contrary to its pathological effects, deregulated activation of PI3K/AKT signaling is also known to play a role in tumorigenesis^19^ and care has to be taken when developing drugs for enhancing cell proliferation and IVD metabolism. The specificity of Phlpp1 on regulating anabolic and apoptotic signaling pathways, which are essential for IVD healing, makes it a promising target for preventing the progression of IDD and its deficiency has a potential to improve cell survival and matrix production in IVDs.

## Materials and methods

### Human lumbar IVDs

Human lumbar IVDs (Thompson grade 1–5) from autopsy were isolated from spinal segments (Fig. 1) by cutting through the vertebrae adjacent to the IVD using a bandsaw (Marmed, Cincinnati, OH, USA). Severity of degeneration of each IVD segment was graded using Thompson scale^36^ prior to fixation in zinc formalin (Z-Fix, Anatech LTD, Battle Creek, MI, USA) and embedding in methyl-methacrylate (Laudier D et al., 2007).

### Mouse model

All animal research was carried out in accordance to recommendations stated in the Guide for the Care and Use of Laboratory Animals of the National Institutes of Health (US Department of Health, Education, and Welfare, NIH 78-23, 1996). All animal protocols were approved by the Mount Sinai Institutional Animal Care and Use Committee (protocol# 2017-0018). Phlpp1 KO mice were obtained from Alexandra Newton’s lab at the University of California, San Diego.

### Mice surgeries

To investigate the effect of Phlpp1 KO on IVD healing, caudal needle puncture injuries were induced in 4–5 months old Phlpp1 KO (*n* = 24) and C57BL/6 J (WT, *n* = 35) mice. Needle punctures are an accepted model to simulate the progression of IDD in vivo^44^. IVD injuries were performed under general anesthesia (2% isoflurane in oxygen) and sterile conditions. Caudal IVDs (cc4-5 and cc6-7, marked by india ink) were identified by palpation and exposed via a 2–4 mm dorsolateral incision. After confirming the location of IVD using a M60 microscope (Leica Microsystems, IL, USA), full AF needle punctures were created using 26 G syringe needles to a depth of 50% of dorsal-ventral width. The needle diameter (26 G, 90% of the IVD height) was chosen based on a previous study^44^. Incisions were sutured (Prolene 8-0 sutures; Ethicon, Somerville, NJ, USA) and mice were closely monitored to ensure the absence of intraoperative complications and allowed free activity in their cages with ad libitum access to food and water. Mice were killed via carbon dioxide inhalation at 3 days, 2 months, and 8 months after surgery.

### Histology and immunohistochemistry

#### Human

Decalcified and methyl-methacrylate embedded human lumbar IVDs were assessed for expression of Phlpp1 and clCasp3. Five-μm thick mid- sagittal sections sections were de-plasticized in a series of xylene, and 2-ethoxyethanol^59^. Following antigen retrieval (Histo/Zyme, H3292, Sigma-Aldrich, St. Louis, MO, USA) and protein-blocking (X0909, DAKO, Carpinteria, CA, USA). IVD sections were incubated with anti Phlpp1 antibody (1:500 dilution, 22789-1-AP, Proteintech) for 1 h at room temperature, followed by incubation with an alkaline phosphatase-conjugated secondary antibody (MP5401, ImmPRESS alkaline phosphatase kit, VECTOR). Samples were then incubated with a red chromogenic staining solution (SK5105, ImmPACT VECTOR Red), counterstained in toluidine blue, and mounted (Eukitt, Sigma-Aldrich, St. Louis, MO, USA). To detect clCasp3, specimens were incubated with anti clCasp3 antibody (Asp175, 1:200 dilution, 9661, Cell Signaling Technology, MA, USA) followed by incubation with a horseradish peroxidase-conjugated secondary antibody (MP6401-15, ImmPRESS VR reagent, Vector Laboratories, Burlingame, CA, USA). Samples were then incubated with a diaminobenzidine-based horseradish peroxidase substrate (SK4105, ImmPACT DAB, Vector Laboratories, Burlingame, CA, USA) to visualize clCasp3 expression. For double staining with Phlpp1, samples were incubated with blocking solution, stained with anti Phlpp1 antibody, and followed the same steps as described above. Normal rabbit serum (NC495, Biocare Medical, Concord, CA) served as a negative control. Samples stained with anti Phlpp1/negative control or anti clCasp3/negative control served as controls for nonspecific double staining. Samples were imaged under bright-field (Phlpp1) or differential interference contrast (Phlpp1/clCasp3 co-expression) microscopy with standardized exposure times (DM6 B automated microscope with LAS X software; Leica Germany).

#### Mice

Decalcified IVD-vertebrae segments were embedded in paraffin and 5-μm-thick mid-sagittal sections were used for histology and immunohistochemistry. IVD morphology was visualized by Picrosirius Red-Alcian Blue staining (PRAB)^60^. Immunohistochemistry was performed for pAkt1 (WT: *n* = 7 per group, Phlpp1 KO: *n* = 5 per group) and aggrecan (*n* = 4 per group at 2 months; *n* = 5 per group at 8 months). For aggrecan, antigen retrieval was performed by applying 0.8% hyaluronidase for 1 h at 37 °C. Following 30 min incubation in blocking solution, samples were incubated for 1 h at room temperature with an anti-aggrecan antibody (1:200 dilution, bs-1223R, Bioss Antibodies Inc, MA, USA). Next, samples were incubated with the secondary, alkaline phosphatase-conjugated secondary antibody (MP5401, ImmPRESS AP reagent, Vector Laboratories) and visualized using alkaline phosphatase substrate (SK5105, ImmPACT Vector Red, Vector Laboratories), counterstained with Mayer’s hematoxylin, and mounted. For pAkt1 staining, samples were incubated for 30 min in blocking solution and incubated with anti pAkt1 antibody (1:100 dilution, 44-621 G, ThermoFisher scientific, MA, USA) for 1 h at room temperature, followed by incubation with the secondary horseradish peroxidase-conjugated antibody (MP6401-15, ImmPRESS VR reagent, Vector Laboratories). pAkt staining was visualized using diaminobenzidine-based horseradish peroxidase substrate (brown, SK4105, ImmPACT DAB, Vector Laboratories), counterstained with toluidine blue, and mounted. Normal rabbit serum (NC495, Biocare Medical) served as negative control for pAkt and aggrecan staining. All Samples were imaged using bright-field microscopy (DM6 B automated microscope with LAS X software; Leica Germany) with standardized exposure times. PAkt immunopositive cells were identified in the AF only (owing to complete NP herniation at day 3) and aggrecan-immunopositive areas were identified in the NP region by calculating the percentage of stained NP tissues, which accumulated in the NP region 2 and 8 months after injury. The immunopositive area and total NP regions were identified by two independent observers who were blinded to experimental groups.

#### Histomorphometric grading scheme

IVD sections of PRAB staining were used to score the degree of degeneration based on histological appearances of the IVD. Sections (for 2 months: control WT *n* = 7, injured WT *n* = 6, control Phlpp1 KO *n* = 10, injured Phlpp1 KO *n* = 8; for 8 months: *n* = 5 per group) were semi-quantitatively graded within eight parameters for signs of degeneration, based on a scoring system published by Tam et al.^33^. One section per IVD was used for analysis. Scores within five categories were added together for a final score out of 14. All sections were examined by two researchers blinded to the experimental groups, and then averaged for analysis.

#### Cell number quantification

Hematoxylin stained mid-sagittal 5-μm thick sections of PRAB staining and immunohistochemistry for aggrecan were used to quantify the cell number in the NP and AF of injured IVDs. Two hematoxylin counterstained sections (one from PRAB staining and one from aggrecan immunostaining) per IVD (2 months: *n* = 7 per group; 8 months: injured WT *n* = 5, injured Phlpp1 KO *n* = 6) were used and the NP and AF areas were used for cell counting using ImageJ. Cell numbers from two hematoxylin counterstained sections were averaged. All sections were examined by two researchers blinded to the experimental groups.

#### EdU assay

To determine the acute response to needle puncture injury, cell proliferation was assessed day 3 post injury (*n* = 7 per group) by EdU assay according to manufacturer’s instructions (ThermoFisher Scientific, Waltham, MA, USA). In brief: EdU (A10044, ThermoFisher Scientific, Waltham, MA, USA) was injected subcutaneously 24 h before sacrifice. Histological IVD-vertebrae sections were stained using an EdU assay kit (C10338, ThermoFisher Scientific, Waltham, MA, USA). One section per IVD was used for analysis. Proliferation was assessed 3 days after injury because this early timepoint should approximately correspond with the end of the initial acute inflammatory response to needle puncture, and the start of the proliferative phase of wound healing.

#### TUNEL assay

Deparaffinized IVD sections (*n* = 4 per group) were washed with PBS then permeabilized using 0.5% Triton-X/PBS (PBST) for 15 min to detect cell death in situ (C10245, ThermoFisher Scientific, Waltham, MA, USA) according to the manufacturer’s instructions. A positive control section was treated with DNase 1 (18068015, ThermoFisher Scientific, Waltham, MA, USA) for 30 min at room temperature and a negative control section was processed by depriving of terminal transferase enzyme. One section per IVD was used for analysis. Images were captured using a Zeiss (Axioimager Z1, Zeiss, Oberkochen, Germany) microscope at × 20 magnification and number of TUNEL-positive cells were counted using the ImageJ interface for selected IVD regions. All sections were examined by two researchers blinded to the experimental groups, and then averaged for analysis.

#### SHG

Unstained paraffin-embedded slides were deparaffinized and sealed to a coverslip with acrylic mount media before imaging and imaged with an Olympus FV1000 MPE laser-scanning microscope (Olympus Corporation, Tokyo, Japan) at the Icahn School of Medicine at Mount Sinai Microscopy Core (for 2 months: control WT *n* = 8, injured WT *n* = 8, control Phlpp1 KO *n* = 6, injured Phlpp1 KO *n* = 7; for 8 months: control WT *n* = 5, injured WT *n* = 5, control Phlpp1 KO *n* = 4, injured Phlpp1 KO *n* = 4). One section per IVD was used for analysis. Tissue sections were excited with a two photon tunable Coherent Chameleon Vision II laser at 910 nm. The SHG emission was collected in the backward direction using the dedicated Olympus WLPLN water-immersive × 25 objective with a numerical aperture of 1.05. This emission was recorded by a photomultiplier tube (PMT) at 440 ± 10 nm to capture the SHG signal at 455 nm. All parameters (i.e., laser intensity, gain, high voltage, and dwell time, aspect ratio) were held constant between 2 and 8 months timepoints to allow comparison of image intensity within each timepoint. To allow for comparison between 2 and 8 months timpoints, the 8 months SHG images were pre-processed to the intensity levels of the 2 months timepoint using a conversion factor determined experimentally by scanning a standard tissue section with the two sets of imaging parameters. All image processing and quantification was performed in Fiji (National Institutes of Health, Bethesda, MD). Maximum intensity z-projection of 10–12 optical sections was performed on mosaic images. The background was subtracted using a 50-pixel rolling ball radius. The posterior AF was manually contoured and the mean grey value was measured to quantify the SHG intensity of the posterior AF and the central NP region.

#### IVD height measurement

DHI was determined 2 months (WT *n* = 18; Phlpp1 KO *n* = 12) and 8 months (WT *n* = 5; Phlpp1 KO *n* = 10) post surgery. Immediately after sacrifice, mice tails were imaged using a high-resolution radiographic system (UltraFocus digital X-ray cabinet, Faxitron Bioptics LLC, AZ, USA). IVD heights and lengths of the adjacent vertebrae were measured using ImageJ. DHI was determined by normalizing the IVD height to the lengths of the adjacent vertebrae using the formula: DHI = 2 × (D + E + F)/(A + B + C + G + H + I) (D−F for IVD heights, A−C,and G−I for anterior and posterior vertebral lengths, respectively).

### Statistical analysis

For statistical analysis, linear regressions were used to evaluate the association of human IDD grade and Casp3 cleavage with Phlpp1 expression. For mice studies, two-way analysis of variances (ANOVAs) were used to assess the effect of injury and genotype. Significant differences between groups were assessed by Bonferroni post hoc testing. Post hoc testing was done to compare injury versus control within each genotype. Differences in healing responses for WT versus Phlpp1 KO were identified from significant interactions in the two-way ANOVA. Power analysis was performed using G*POWER to determine the sample size (alpha is 0.05 and power is 0.80). Statistical analyses were performed using Graphpad Prism7 (GraphPad Software, Inc., La Jolla, CA). A *p* value < 0.05 was considered statistically significant.
